# Steroid receptor coactivator-1 interacts with NF-κB to increase VEGFC levels in human thyroid cancer

**DOI:** 10.1042/BSR20180394

**Published:** 2018-06-12

**Authors:** Bo Gao, Lingji Guo, Donglin Luo, Yan Jiang, Jianjie Zhao, Chengyi Mao, Yan Xu

**Affiliations:** 1 Department of Breast and Thyroid Surgery, Research Institute of Surgery of Daping Hospital, Army Military Medical University, Chongqing 400042, China; 2 Department of Pathology, Research Institute of Surgery of Daping Hospital, Army Military Medical University, Chongqing 400042, China

**Keywords:** lymphatic metastases, SRC-1, thyroid cancer, VEGFC

## Abstract

Thyroid cancer is the most common endocrine cancer, and has a high incidence of lymphatic metastasis. Vascular endothelial growth factor C (VEGFC) is essential for development of lymphatic vessels and lymphatic metastases during carcinogenesis. Steroid receptor coactivator-1 (SRC-1) interacts with nuclear receptors and transcription factors to promote tumor proliferation and metastasis. However, the correlation between SRC-1 and VEGFC levels in the lymphatic metastases of thyroid cancer remains unclear. We analyzed 20-paired specimens of thyroid cancer tissue and normal thyroid tissue and found increased levels of SRC-1 and VEGFC proteins in 13/20 and 15/20 thyroid cancer specimens, respectively, when compared with those levels in specimens of normal thyroid tissue. A high level of SRC-1 expression was positively correlated with VEGFC and lymphatic endothelial cell marker LYVE-1 expression. Papillary thyroid carcinoma cell line TPC-1 displayed high levels of SRC-1 and VEGFC expression and was selected for stable knockdown of *SRC-1 in vitro*. Inhibition of SRC-1 significantly reduced the VEGFC levels in TPC-1 cells. We found that SRC-1 binds to transcription factor NF-kB (p50/p65), and that this coactivation complex directly promoted *VEGFC* transcription, which could be abrogated by *SRC-1* knockdown. Up-regulated NF-kB signaling was also confirmed in thyroid cancer tissues. *In vivo* studies showed that *SRC-1* knockdown restricted tumor growth, reduced the numbers of LYVE-1-positive lymphatic vessels, and decreased the levels of VEGFC in tumor tissues. These results suggest a tumorigenic role for SRC-1 in thyroid cancer via its ability to regulate VEGFC expression.

## Introduction

Thyroid cancer is the most common type of endocrine cancer and the sixth most common cancer in women. More than 62,000 new cases of thyroid cancer were diagnosed in men and women during 2015, and its incidence continues to rise worldwide [1]. Differentiated papillary and follicular thyroid carcinomas are the most commonly diagnosed subtypes and have the best overall prognosis. In contrast, the patients with anaplastic thyroid carcinoma have a very poor prognosis [1,2]. Because the 10-year survival rate among patients with papillary thyroid cancer is 93%, therapeutic advances have been minimal during the past two decades [3]. Thyroid cancer commonly metastasizes to regional lymph nodes in the central and lateral compartments of the neck, and these metastases affect the patient’s prognosis. The patients who have a papillary thyroid cancer with macroscopic metastases (e.g. >3 cm extra nodal extension) in their cervical lymph nodes have higher rates of disease recurrence and cancer-related death, and this is especially true among patients aged 45 years and older [1,4].

The development of new lymphatic vessels (lymphangiogenesis) is essential for tumor cells to migrate into lymph nodes (i.e. lymphatic metastasis) [5,6]. Vascular endothelial growth factor C (VEGFC) plays a prominent role in the lymphatic metastasis of many cancers, including gastric cancer [7], cervical cancer [8], melanoma [9], and thyroid cancer [10]. Yu et al. [11] reported that VEGFC is overexpressed in thyroid cancers, and this overexpression correlates with lymph node metastases [11]. VEGFC binds to its receptor VEGFR3 and also interacts with various pathway-related molecules such as lymphatic endothelial cell marker LYVE-1, SDF-1, and its receptor CXCR4, to induce the proliferation of lymphatic endothelial cells *in vitro* and lymphangiogenesis *in vivo* [12,13]. However, the molecular mechanisms of VEGFC-induced lymphangiogenesis are not fully known in thyroid cancer.

Steroid receptor coactivator 1 (SRC-1, also known as NCOA1) was first discovered in 1995 by investigators who used a yeast two-hybrid screening system, and was identified as a global nuclear receptor coactivator. However, the steroid-independent role of SRC-1 has now been recognized, and this coactivator has been demonstrated to interact with other transcription factors including NF-kB, AP-1, Ets2, and PEA3 [14,15]. *SRC-1* is a tumorigenic gene that is critical for enabling endocrine tumors to adapt and become resistant to targeted therapies. *SRC-1* has been shown to promote cellular proliferation and the metastasis of breast cancer [16], hepatocellular carcinoma (HCC) [17], astrocytoma [18], and thyroid cancer [19]. In nonanaplastic thyroid cancer, SRC-1 expression is associated with poorly differentiated tumors, capsular invasion, and disease recurrence [19].

In the present study, we revealed how SRC-1 promotes tumorigenesis by participating in VEGFC-induced lymphangiogenesis in cases of thyroid cancer. The levels of SRC-1 expression in samples of thyroid cancer tissue, and their correlation with VEGFC levels and LYVE-1-positive lymphatic vessels were analyzed. The regulatory mechanism of SRC-1/VEGFC was determined *in vitro* and the role played by SRC-1 in thyroid cancer was investigated *in vivo*.

## Materials and methods

### Tumor samples, cell lines, and reagents

Twenty specimens of thyroid cancer tissue and corresponding normal thyroid tissue were collected at Daping Hospital of Army Military Medical University (Chongqing, China) and analyzed for their levels of mRNA for SRC-1, VEGFC, and LYVE-1 by use of immunohistochemistry, Western blotting, and Q-PCR. The thyroid cancer tissues included ten papillary carcinomas, five follicular adenomas, three medullary cancers, and two anaplastic cancers. For statistical purposes, all thyroid cancers were grouped as a single type. All diagnoses of primary thyroid cancer were confirmed according to the WHO classification system by experienced pathologists who examined the tissue specimens after staining with hematoxylin and eosin. The study protocol was approved by the Research Ethics Committee of Daping Hospital of Army Military Medical University, and all tissue specimens were processed and analyzed in an anonymous manner according to ethical and legal standards. Each cancer patient provided their written informed consent for study participation.

Normal human thyroid cell line Nthy-ori 3-1 and seven human thyroid carcinoma cell lines (WRO, FTC-133, B-CPAP, SW579, and TPC-1) were purchased from the cell bank of the Chinese Academy of Sciences (Shanghai, China) and cultured in either Dulbecco’s Modified Eagle’s medium or 1640 medium supplemented with 10% FBS (Life Technologies, Carlsbad, CA, U.S.A.), ampicillin, and streptomycin at 37°C in a 5% CO_2_ atmosphere. A lentivirus containing shRNA-SRC-1 or an NC vector was prepared by GenePharma (Shanghai, China) and used for knockdown of *SRC-1*. A virus containing the pcDNA3.1-p65 vector was produced by GenePharma (Shanghai, China) and used to force overexpression of p65. A reporter plasmid of the full-length promoter construct (wild-type or mutant) of pGL-3-VEGFC was prepared by GenePharma (Shanghai, China). Anti-SRC-1, VEGFC, LYVE-1, p65, p50, and GAPDH antibodies were obtained from Cell Signaling Technology (Danvers, MA, U.S.A.) and Abcam (Cambridge, MA, U.S.A.).

### Western blot studies

The total proteins were extracted from tumor or normal tissues and separated on a 10% SDS polyacrylamide gel. Next, the separated protein bands were transferred onto a nitrocellulose membrane, which was then blocked with 5% BSA in TBST buffer (Tris buffer saline containing 0.1% Tween-20) for 1 h at room temperature. After blocking, the membrane was incubated with anti-GAPDH, SRC-1, and VEGFC antibodies overnight at 4°C. GAPDH was used as a loading control in the Western blot studies. After washing with TBST buffer, the blots were incubated with an HRP-conjugated secondary antibody for 1 h at room temperature. After washing with TBST buffer, the blots were visualized with ECL-Plus reagent (Millipore, Billerica, MA, U.S.A.).

### RNA isolation and qRT-PCR

Total RNA was extracted from TPC-1 cells or tumor tissues using Trizol reagent (Invitrogen, Carlsbad, CA, U.S.A) according to the standard RNA isolation protocol. Quantitative real-time RT-PCR (qRT-PCR) was performed, and the expression levels of *SRC-1, VEGFC, LYVE*-1, and *NF-κB* were normalized to the level of *GAPDH* gene expression. The primers used for qRT-PCR are shown in Table 1.

**Table 1:** The primers of VEGFC, SRC-1, LYVE-1, and NF-κB

ID	Sequence (5′- 3′)	Product length (bp)
GAPDH F	TGTTCGTCATGGGTGTGAAC	154
GAPDH R	ATGGCATGGACTGTGGTCAT	
VEGFCF	GAGCAGTTACGGTCTGTGTCCA	101
VEGFCR	TGCCAGCCTCCTTTCCTTAGC	
SRC-1 F	CTCATGGTGTGGCTCGTTCATC	80
SCR-1 R	GCTCTGCTGGCGGTTTATTCTG	
LYVE-1 F	TTTCCATCCAGGTGTCATGC	262
LYVE-1 R	TTCCAAATCAGGACACCCAC	
NF-κB F	TCCTATAGAAGAGCAGCGTG	231
NF-κB R	TGCACCTTGTCACACAGTAG	

### Immunohistochemistry

The relative levels of SRC-1, VEGFC, LYVE-1, and NF-κB protein expression in HCC tissues were examined in 2-μm-thick, formalin-fixed, paraffin-embedded tissue specimens. Immunohistochemistry assays were performed as previously described [20]. The slides with tissue sections were first incubated in xylene for 5 min, and then incubated in 100% ethanol for 10 min followed by 95% ethanol for 10 min. Antigen unmasking was performed and the slides were then blocked with 3% hydrogen peroxide for 30 min at room temperature. The FFPE tissue specimens were then incubated with primary antibodies for SRC-1, VEGFC, LYVE-1, and NF-κB at 4°C overnight, following by incubation with a biotinylated horse secondary antibody and streptavidin-horse-radish peroxidase (Zymed Laboratories Inc., South San Francisco, CA, U.S.A.). An EnVision Detection System kit (DAKO, Denmark) was used for the DAB chromogen followed by nuclear staining with hematoxylin.

### Cell transfection

Cell transfection studies were performed to estimate the interaction between NF-kB and the *VEGFC* promoter region. TPC-1 cells were seeded into 12-well plates; after which, Lipofectamine 2000 (Invitrogen) was used to transfect the cells with the indicated agents for the specified time period at a concentration of 1 ng/ml according to the manufacturer’s instructions.

### Luciferase reporter assay

TPC-1 cells were co-transfected with pcDNA3.1-p65 and the reporter plasmid of the full-length promoter construct (wild-type or mutant) of pGL-3-VEGFC or an NC plasmid. Luciferase activity was measured using a Dual-Luciferase Reporter Assay System (Promega, Madison, WI, U.S.A.). Firefly luciferase served as a reporter gene for the normalized control.

### Electrophoretic mobility shift assay

The DNA-binding activity of NF-kB was determined by the electrophoretic mobility shift assay (EMSA). The EMSAs were performed on nuclear extracts prepared from different groups of cells and using a nuclear extract kit (Active Motif; Rixensart, Belgium) according to the manufacturer’s instructions. The related probes were synthesized (Invitrogen) and incubated with nuclear extracts contained in the reaction solution. Nonlabeled wild-type and mutant double-stranded competitor oligonucleotides were added to the respective reactions. Binding specificity was examined by competition with the nonlabeled self, mutant, and consensus oligonucleotides.

### Co-immunoprecipitation (CO-IP) assay

TPC-1 cells were harvested and crosslinked with 1% formaldehyde at room temperature. After sonication and removal of cellular debris, 2 mg of anti-SRC-1 mAb plus 10 ml of Protein G Sepharose slurry was added to the cell lysate solution, and the mixture was incubated overnight at 4°C. SRC-1 immunoprecipitates (IPs) were washed three times. The proteins were then released from the beads by heating with Laemmli buffer, and analyzed by SDS-PAGE and Western blotting methods. Precipitated proteins were detected with anti-p65, anti-p50, and anti–SRC-1 mAbs, respectively.

### Tumor model

To test the role of SRC-1 *in vivo*, TPC-1 cells were transfected with lentivirus containing shRNA-SRC-1 or an NC vector. The transfected TPC-1 cells (2 × 10^6^) were subcutaneously injected into the rear flanks of nude mice (six mice per group). The tumor sizes were measured at 3-day intervals and the tumor volumes were calculated as follows: V (cm^3^) = width^2^ (cm^2^) × length (cm) / 2. The mice were killed on day 26.

### Statistical analyses

All results were analyzed using the SPSS Statistical Package for Social Sciences, Version 16.0 (SPSS Inc., Chicago, IL, U.S.A.) and the Prism statistical software package (Version 5.0, Graphpad Software Inc.). Unpaired *t*-tests or Mann–Whitney *U* tests were used for comparisons between two groups, and one-way ANOVA was used for multiple group comparisons. *P-*values < 0.05 were considered statistically significant. All experiments were performed at least three times.

## Results

### Expression of SRC-1 was positively correlated with VEGFC in thyroid cancer

**Figure 1:**
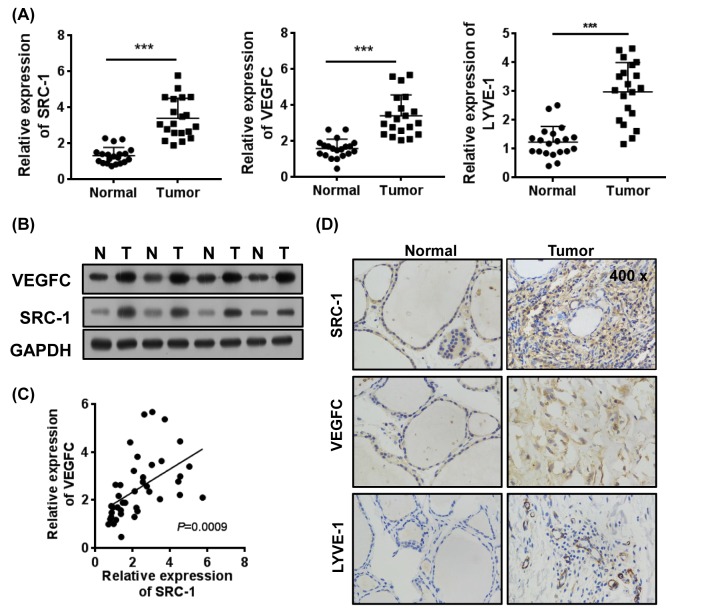
The expression of SRC-1 positively correlates to VEGFC in thyroid cancer (**A**) The levels of mRNA expression for SRC-1, VEGFC, and LYVE-1 in thyroid cancer and relatively normal tissues (*n*=20) were determined by Q-PCR. (**B**) The levels of SRC-1 and VEGFC proteins in tumor tissues were determined by Western blot. (**C**) The correlations between SRC-1 and VEGFC in thyroid cancer tissues were analyzed. (**D**) The expression patterns of SRC-1, VEGFC, and LYVE-1 in thyroid cancer and relatively normal tissues were confirmed by immunohistochemistry. ***P<0.001; data represent the mean ± S.D.

We collected 20-paired samples of thyroid cancer tissue and corresponding normal tissue and used them to investigate the clinical patterns of SRC-1 expression in thyroid cancer. Our Q-PCR and Western blot analyses showed that SRC-1 expression was significantly up-regulated in the thyroid cancer tissues (Tumor) when compared with expression in the corresponding non-neoplastic tissues (Normal). Moreover, the levels of VEGFC and lymphatic endothelial cell marker LYVE-1 expression were also up-regulated in the cancer tissues (Figure 1A,B). An analysis of expression levels identified a positive correlation between SRC-1 and VEGFC levels in the thyroid cancer tissues, and showed that SRC-1 levels were positively associated with VEGFC expression in the tumors (*r*^2^ = 0.6756; 95% CI: 0.4536–0.8187) (Figure 1C). Expression of the lymphatic endothelial cell marker LYVE-1 was also correlated with a high level of SRC-1 expression in thyroid cancer tissue. Furthermore, an immunohistochemistry analysis confirmed that the proteins SRC-1/VEGFC (positive: 13/20 and positive: 15/20) and SRC-1/LYVE-1 (positive: 13/20 and positive: 18/20) were highly expressed in a majority of thyroid cancer specimens (Figure 1D). These data suggest that high levels of SRC-1 might facilitate the up-regulation of VEGFC expression in thyroid cancer tissue.

### Knockdown of *SRC-1* in thyroid carcinoma cell line TPC-1 inhibited VEGFC expression

**Figure 2:**
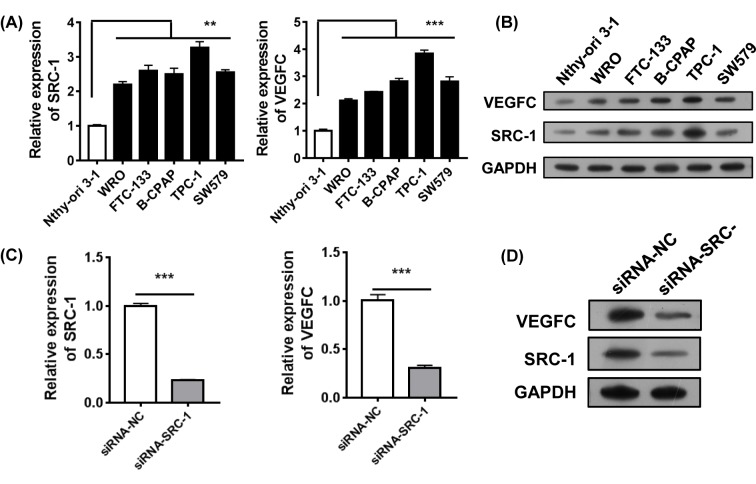
Knockdown of SRC-1 inhibited VEGFC expression *in vitro* (**A** and **B**) The levels of SRC-1 and VEGFC expression in normal human thyroid cell line Nthy-ori 3-1 and seven human thyroid carcinoma cell lines (WRO, FTC-133, B-CPAP, SW579, and TPC-1) were analyzed by Q-PCR and Western blot. (**C** and **D**) After knockdown of *SRC-1* in TPC-1 cells, the levels of SRC-1 and VEGFC were estimated by Q-PCR and Western blot. **P<0.01, ***P<0.001; data represent the mean ± S.D.

We next investigated how SRC-1 might help regulate VEGFC expression *in vitro*. Normal human thyroid cell line Nthy-ori 3-1 and five human thyroid carcinoma cell lines (WRO, FTC-133, B-CPAP, SW579, and TPC-1) were analyzed for their SRC-1 mRNA and protein levels. The results showed that papillary thyroid carcinoma cell line TPC-1 had the highest level of SRC-1 expression (Figure 2A,B). Furthermore, the levels of VEGFC were also highest in the TPC-1 cells. Thus, TPC-1 cells were selected as model in which to study the effects of steady knockdown of *SRC-1* by lentivirus-shRNA. The efficiency of knockdown was confirmed. Our data showed that expression of SRC-1 in the TPC-1/shRNA-SRC-1 cells was strongly inhibited when compared with SRC-1 expression in the TPC-1/shRNA-NC cells. Similarly, the levels of VEGFC were also decreased by the *SRC-1* knockdown (Figure 2C,D).

### Co-activation complex SRC-1/NF-kB directly promoted *VEGFC* transcription

**Figure 3:**
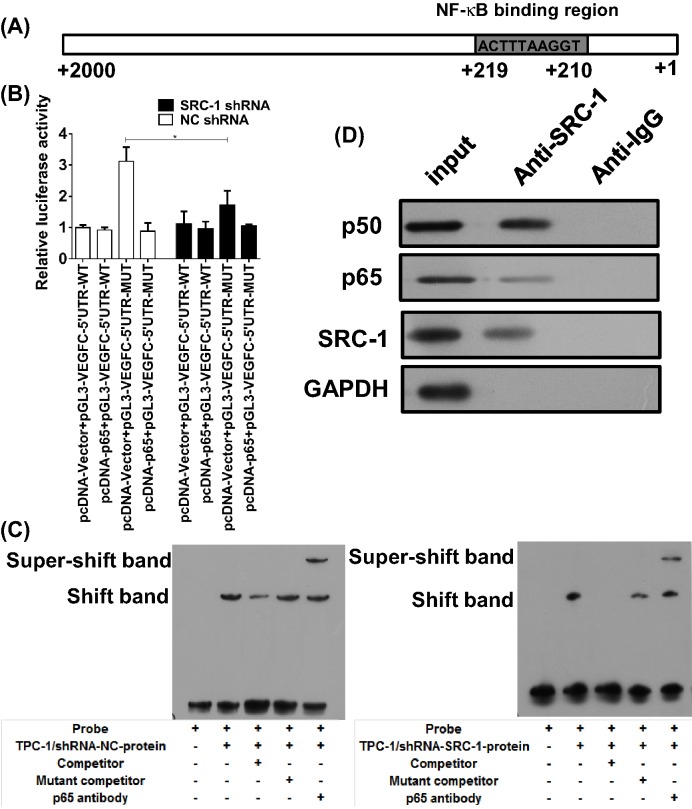
SRC-1 interacts with NF-kB for transcription of *VEGFC* (**A**) The predicted p65 binding sites in the promoter region of *VEGFC*. (**B**) A luciferase reporter vector with the full-length promoter construct of VEGFC containing mutations at p65 binding sites was transfected into TPC-1/shRNA-SRC-1 or shRNA-NC cells, and the luciferase activity was determined. (**C**) Biotin-labeled oligonucleotide probes corresponding to the *VEGFC* promoter formed complexes with p65 in the presence or absence of anti-p65 antibodies or specific/mutant competitors. These complexes were detected by EMSA. (**D**) The SRC-1/NF-kB (p50/p65) complexes in TPC-1 cells were precipitated by an anti-SRC-1 antibody, and the p50/p65 levels were determined. *P<0.05; data represent the mean ± S.D.

Because we found that VEGFC levels could be reduced by *SRC-1* knockdown, we speculated that SRC-1 might interact with other transcription factors to form a co-activation complex that induces transcription of the *VEGFC* gene. A bioinformatics analysis revealed that the human *VEGFC* promoter region harbors putative binding sequences for NF-kB (p65/p50), and one predicted binding site was identified (Figure 3A). We next performed luciferase reporter assays to confirm the NF-kB binding site within the VEGFC promoter region in ovarian cancer cells. A luciferase reporter vector with the full-length promoter construct of VEGFC, but containing mutations at the NF-kB binding sites was transfected into TPC-1 cells. The results showed that the luciferase activity of the wild-type promoter construct of VEGFC could be enhanced by NF-kB overexpression, which was negative in the mutant-type promoter construct of VEGFC (Figure 3B). Interestingly, the increase in luciferase activity was significantly diminished in TPC-1/shRNA-SRC-1 cells, indicating that knockdown of *SRC-1* had suppressed the effect of NF-kB on VEGFC. Next, oligonucleotide probes with mutations at the VEGFC motif sites were synthesized for EMSA analysis. The results further confirmed that NF-kB binds directly to the VEGFC promoter to enhance *SRC-1* transcription in TPC-1 cells. This enhancement was impaired by *SRC-1* knockdown (Figure 3C).

We next analyzed the relationship between SRC-1 and NF-kB in TPC-1 cells. Because SRC-1 as a co-activator has been demonstrated to interact with several transcription factors including NF-kB, we speculated that SRC-1 might interact with the NF-kB (p65/p50) complex to form a co-activation complex. An immunoprecipitation assay performed with anti-p65 and p50 antibodies demonstrated that SRC-1 binds directly to the NF-kB (p65/p50) complex in TPC-1 cells. These results suggest that SRC-1 interacts with NF-kB (p65/p50) to form a co-activation complex that induces transcription of the *VEGFC* gene in TPC-1 cells (Figure 3D).

### Increased NF-kB signaling in thyroid cancer tissues

**Figure 4:**
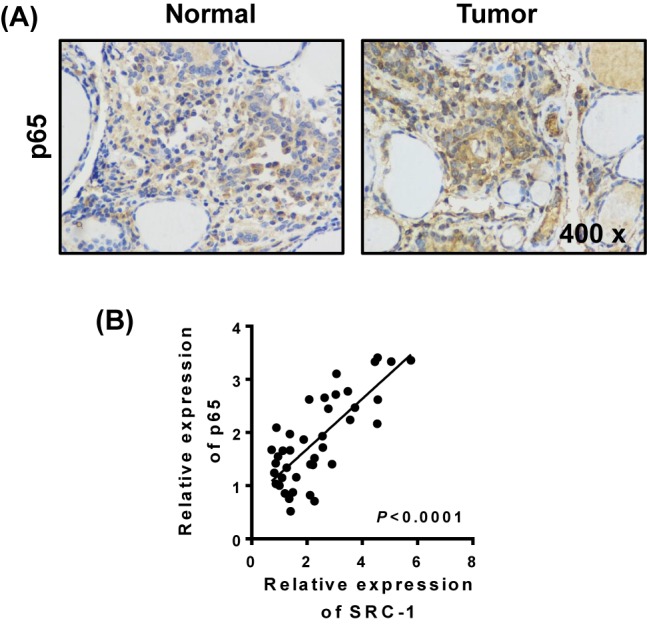
Increased NF-kB signal in thyriod cancer tissues The levels of p65 expression in thyroid cancer and relatively normal tissues (*n*=20) were determined by (**A**) immunohistochemistry. (**B**) The correlation between p65 and SRC-1 was analyzed by Q-PCR. *P* < 0.001; data represent the mean ± S.D.

Because we had previously demonstrated that NF-kB is required for SRC-1-induced VEGF expression, we next investigated the clinical expression of NF-kB signaling in thyroid cancer tissue. An immunohistochemistry analysis showed that p65 expression was significantly up-regulated in thyroid cancer tissues when compared with p65 expression in the corresponding non-neoplastic tissues (Figure 4A). Furthermore, this increase in p65 levels was positively correlated with SRC-1 expression in thyroid cancer tissue (*r*^2^ = 0.8163; 95% CI: 0.5392–0.9086) (Figure 4B).

### Knockdown of *SRC-1 in vivo* inhibited tumor growth and reduced lymphangiogenesis

**Figure 5:**
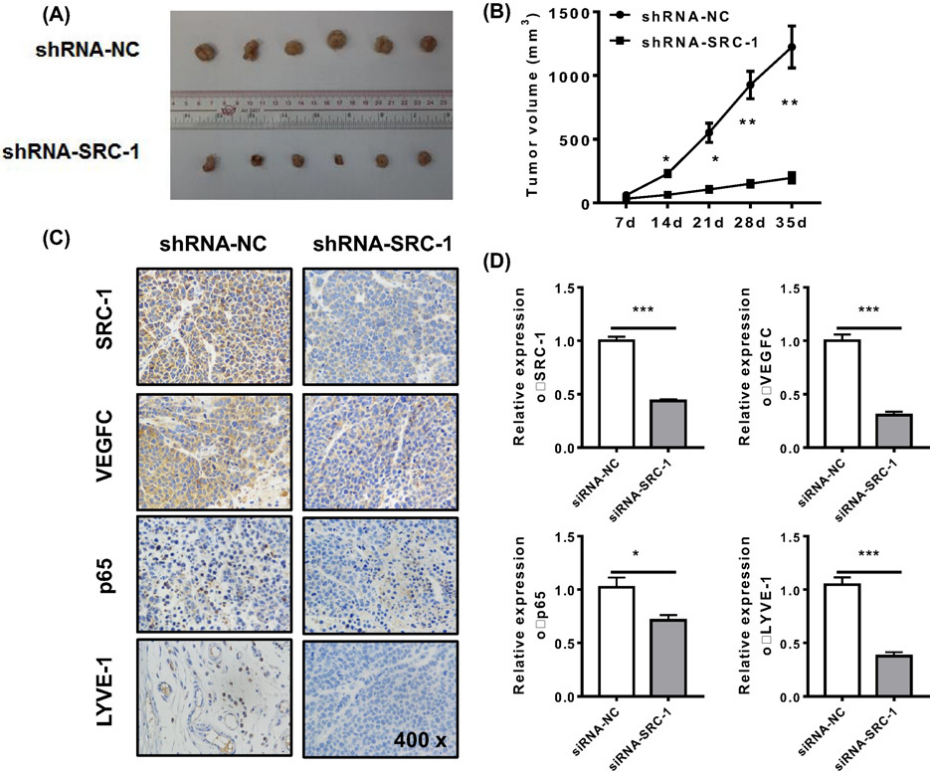
The tumorigenic role of SRC-1 was assessed *in vivo*. TPC-1/shRNA-SRC-1 or shRNA-NC cells (2 × 10^6^ per cell type) were subcutaneously injected into the rear flanks of nude mice (six mice per group). (**A** and **B**) The mean tumor size in each group (mm^3^) was analyzed. (**C** and **D**) The levels of SRC-1, VEGFC, p65, and LYVE-1 expression were estimated by immunohistochemistry and Q-PCR. *P<0.05, **P<0.001, ***P<0.001; data represent the mean ± S.D.

To provide *in vivo* evidence for the tumorigenic role of SRC-1, we injected nude mice with equal numbers of TPC-1/shRNA-SRC-1 or shRNA-NC cells. We found that the mice injected with TPC-1/shRNA-SRC-1 cells displayed delayed tumor growth (Figure 5A) and smaller the tumor volumes (Figure 5B) when compared with the mice injected with TPC-1/shRNA-NC cells. The numbers LYVE-positive lymphatic vessels in the mice treated with TPC-1/shRNA-SRC-1 cells were significantly lower than those numbers in the control groups. The mRNA levels for SRC-1, VEGFC, p65, and LYVE-1 were also reduced by *SRC-1* knockdown *in vivo* (Figure 5C,D). These data suggest that knockdown of *SRC-1 in vivo* can inhibit VEGFC expression and reduce lymphangiogenesis in thyroid cancer.

## Discussion

The numbers, structures, and functions of lymphatic endothelial cells affect lymphangiogenesis, which is essential for tumor lymphatic metastasis. The VEGFC/D-VEGFR3/NRP2 axis is the pathway responsible for the formation and reconstruction of pre-existing and newborn lymphatic vessels [12]. Therefore, it is extremely important to understand the regulatory mechanism of VEGFC-related signals. In the present study, we found that the steroid receptor co-activator (SRC)-1 was up-regulated in thyroid cancer tissue. This up-regulated SRC-1 then interacts with transcription factor NF-kB to promote transcription of the *VEGFC* gene, leading to increased VEGFC levels and lymphangiogenesis. Other molecules have also been reported to control the VEGFC levels in cancer. Liu et al. [21] reported that transducin (β)-like 1 X-linked receptor 1 induces lymphangiogenesis and lymphatic metastasis in esophageal squamous cell carcinoma via up-regulation of VEGF-C. Sine oculis homeobox homolog 1 (SIX1) was found to enhance activation of the TGF-β pathway, and thereby increase VEGF-C expression to the levels needed for lymph node metastasis [22]. Furthermore, the TNF-α–TNFR1 signalling pathway has been shown to regulate the VEGFC expression [23].

The steroid receptor co-activator (SRC) family comprises three homologous members (SRC-1, SRC-2, and SRC-3) that interact with nuclear receptors and numerous transcription factors to enhance target gene transcription. Dysregulation of SRCs is strongly implicated in many cancers, and especially in hormone-dependent cancers [19]. SRC-1 expression was found to be significantly increased in breast cancers, and positively correlated with ERBB2 expression, disease recurrence, and poor survival rates [24]. SRC-3 was found to be overexpressed in prostate cancer, and was positively associated with a high tumor grade and disease recurrence [25]. Tong et al. [17] reported that SRC-1 was overexpressed in 25 (62.5%) of 40 human HCC specimens [17]. However, few studies have investigated SRC-1 expression in thyroid cancer. Kavanagh et al. [11] reported that only a low percentage of papillary and follicular tumors, but a high percentage of anaplastic tumors (87%) express SRC-1. In this study, we found that the SRC-1 expression in samples of thyroid cancer tissue was elevated when compared with SRC-1 expression in the corresponding normal tissues, and 26/40 samples expressed SRC-1.

A study conducted with MMTV-PyMT (mouse mammary tumor virus-polyoma middle T) transgenic mice showed that *SRC-1* knockout significantly inhibited mammary tumor cell intravasation and metastasis to the lungs, and reduced the numbers of circulating tumor cells in the blood [26]. Here, we found a correlation between high levels of SRC-1 expression and increased numbers of LYVE-positive lymphatic vessels. Moreover, the *in vivo* knockdown of *SRC-1* in thyroid cancer reduced the numbers of LYVE-positive lymphatic vessels in mice, indicating the involvement of SRC-1 in lymphangiogenesis. Many molecular and *in vivo* studies have revealed mechanisms in which SRC-1 serves as a co-activator for certain transcription factors (e.g. AP-1 and PEA3) that activate tumorigenic genes (i.e. *twist, integrin-a5, ADAM22*, and *c-Myc*) known to promote disease progression and metastasis [14,16,27]. In L929 cells, SRC-1 specifically binds to the p50 subunit, but not to the p65 subunit of transcription factor NF-kB, as demonstrated by the yeast two-hybrid test and glutathione S-transferase pull down assay. Similarly, we found that in thyroid cancer, SRC-1 interacts with NF-kB (p65) to form a co-activation complex that induces transcription of the *VEGFC* gene in TPC-1 cells. Knockdown of *SRC-1* in thyroid cancer *in vivo* inhibited VEGFC expression, which led to a reduction in lymphangiogenesis. This finding indicates that SRC-1 regulates VEGFC expression and participates in tumor metastasis via NF-kB, which is consistent with results in a previous study conducted with human astrocytoma cell lines [28]. In that study, activation of progesterone/progesterone receptor (PR) signaling significantly increased VEGF and EGFR mRNA expression, and while SRC-1 was not required for PR-mediated EGFR mRNA transcription, it was needed for VEGF mRNA transcription.

In conclusion, we have identified a new mechanism for the processes of lymphangiogenesis and lymph node metastasis. We found that SRC-1 was up-regulated in thyroid cancer tissues and interacted with NF-kB signals to promote VEGFC expression and increase the numbers of LYVE-positive lymphatic vessels. *In vivo* knockdown of SRC-1 represents a promising strategy for reducing or preventing thyroid cancer metastasis.

## Abbreviations

EMSA: electrophoretic mobility shift assay
HCC: hepatocellular carcinoma
IP: immunoprecipitate
PR: progesterone receptor
qRT-PCR: quantitative real-time RT-PCR
SRC: steroid receptor co-activator
VEGFC: vascular endothelial growth factor C
